# Emotional reactions and stigmatization after a parricide in South Tyrol, Italy, among mental health professionals and the general population, including persons with mental disorders, relatives, and persons with no direct or indirect contact

**DOI:** 10.3389/fpubh.2024.1388842

**Published:** 2024-07-01

**Authors:** Mara Stockner, Anna Wenter, Artur Obexer, Isabella Gualtieri, Francesca Merler, Davide Bennato, Andreas Conca

**Affiliations:** ^1^Department of Dynamic and Clinical Psychology, Faculty of Psychology, Sapienza University of Rome, Rome, Italy; ^2^Department of Psychology, Institute of Psychology and Sports, University of Innsbruck, Innsbruck, Austria; ^3^Department of Psychiatry, Health District of Bolzano (SABES-ASDAA), Bolzano, Italy; ^4^Department of Humanities, University of Catania, Catania, Italy

**Keywords:** parricide, emotional reactions, stigmatization, mental health professionals, relatives

## Abstract

**Introduction:**

This study was conducted on the occasion of the parricide in Bolzano (South Tyrol, Italy) in January 2021. The psychological impact of parricide on the general population and on mental health professionals has scarcely been investigated to the present day. Studies on stigmatization show differences between various groups. The aim was to analyze the emotional reactions to the parricide and the stigmatization of persons with mental disorders in the South Tyrolian population.

**Methods:**

In September 2022, 121 mental health professionals of the Department of Psychiatry in Bolzano were surveyed using an online questionnaire. In addition, from January to March 2023, the general population of South Tyrol was invited to take part in the survey through an online-link and was divided into three groups: 267 persons with mental health problems, 855 relatives and 1,019 persons with no direct or indirect contact to people with mental problems. The validated Reported and Intended Behavior Scale (RIBS) was used together with questions on the emotional reactions to the parricide and the perceived dangerousness of psychiatric patients. Descriptive statistics, one-way Anovas as well as regressions were carried out.

**Results and discussion:**

All groups experienced sadness the most. Relatives experienced more sadness and anger than the other groups. Over 80% of the professionals stated that psychiatric patients were not at greater risk of committing parricide. The population with no contact rated the risk higher than those affected and had the lowest level of openness (RIBS). There were no differences between genders, but there were age differences, with younger people being more stigmatizing. The results suggest that personal contact, appropriate information, and education are associated with less stigmatization.

## 1 Introduction

The present study was conducted on the occasion of the parricide in Bolzano (South Tyrol, Italy) on 4th January 2021, in which a young man killed both his parents (1). The 31-years-old man killed his parents, both retired teachers, with an alpinist rope at the family home (2). The parents had been reported missing by their son and were searched for weeks (3). The mother’s body was found 1 month later, the father’s almost 4 months later—both in a local river (2). The perpetrator only confessed to the murder after the mother’s body was found (4). In November 2022, he was found guilty of both murders and sentenced to life imprisonment (5). Before the parricide, he had been hospitalized in a psychiatric clinic in Germany in the summer 2020 (diagnosis: personality disorder, paranoid schizophrenia) and he had also consulted various psychiatrists and therapists in South Tyrol (6), including a short hospitalization in a psychiatric unit.

Parricide (i.e., the murder of one’s own parents) is a universal theme that runs through all cultures and times (7–9). The mythical stories of Oedipus’ patricide and Orestes’ matricide have created an archetype that is widespread in many religions and cultures of the West. It forms the basis for contemporary psychiatric and psychoanalytic approaches to understanding violence against parents and parricide (9). Regardless of society, culture, or religion, it is considered inherently evil to kill others, especially one’s own parents (10).

Parricides have always attracted the attention of the media in all cultures and continue to shock the public (11). The mass media tend to portray these acts of violence as sensational (9, 11). The scandalous nature of parricide has long attracted public fascination (12). The parricide in Bolzano in 2021 attracted a great deal of media coverage, too. The case filled countless newspaper pages and programs, both at local and national level. Like a TV thriller, every little detail was distorted and linked together (13). Some of the most notable headlines, portraying the perpetrator as evil and mental ill, were: “Böse, nicht krank” [“Evil, not sick”] (5), “Hatten alle Angst vor Benno” [“They were all afraid of Benno”] (14), “Benno Neumair, la difesa: ‘È malato, non è imputabile’” [“Benno Neumair’s defense: ‘He is ill, he is not imputable’”] (15), “Prozesstag 1: ‘Benno Neumair ist krank, er hat eine besonders schwere Störung’” [“On the first day of the trial: ‘Benno Neumair is ill, he has a particularly severe disorder’”] (16), “Benno hat es sich nicht ausgesucht, krank zu sein” [“Benno did not choose to be ill”] (17), “Benno Neumair, la maestra d’asilo: ‘Andava curato da piccolo e la madre lo portò da uno stregone’”[“Benno Neumair, the kindergarten teacher: ‘He should have been cured as a child and his mother took him to a witch doctor’”] (18). In German media he was labeled as “wohl bekanntester Doppelmörder in Italien” [“probably the most famous double murderer in Italy”] (19).

Worldwide and in Italy, parricide accounts for about 3–4% of all solved homicides and is therefore a rare event (20–22). In Italy, 40.1% of the cases occurred in the North (where South Tyrol is located), 28.4% in the South, 16.1% in the Centre and 15.4% on the islands (20). In South Tyrol, the following picture emerges with regard to parricide: in 1985 a man killed his father and his brother (23), in 1987 a young man killed his 47-years-old mother (24), in 2002 a 20-years-old man killed both his parents (25) and in 2016 a 45-years-old man killed his 87-years-old mother (26). The murder in Bolzano in 2021 is the only parricide in South Tyrol’s more recent history in which both parents were killed by their son. The literature on parricide is sparse, the evidence base is limited, and most studies have a small sample, making generalization difficult (12, 22). Most studies refer to Western contexts (10). For example, there are studies on the alleged causes, judicial outcomes, and media portrayal of parricides [e.g., (7, 27, 28)]. The criminological focus predominates (9): parricide is a crime committed mainly by men (22, 29); they are most often unmarried, unemployed (29) and often still live at home with their parents (12). Regarding the correlation between parricide and mental disorders, Melo and Alves (22) stated that a large proportion but not all adult parricidal offenders suffer from a psychiatric disorder. Bojanić et al. (29) found that one third of the offenders were diagnosed with schizophrenia; 28% had been in contact with mental health services before the offense. A Turkish study on 135 adult perpetrators of parricide (30) found that 58.5% were not diagnosed with mental disorders, while psychotic disorders were identified in 30.4% of the cases.

Grattagliano et al. (31) stated that parricide has a strong emotional impact on public opinion. Public opinion on parricide is a combination of fascination and horror (12). However, the psychological impact of parricide on the general population and mental health professionals, and the stigmatization of mental health problems in the context of parricide have not, to our knowledge, been explored. We know from theories and research on criminality (not necessarily parricide) that criminality is associated with emotions such as fear and anger (32). Inflated, consistent and continuous levels of crime reporting in media contribute to a relative high level of concern and fear (33). In fact, the general public often comes into contact with crime [e.g., (34)] and mental illness [e.g., (35)] through the media. In general, the media contain negative statements that reflect stigmatization of people with mental illness (36). People with mental health problems are often portrayed as unpredictable, violent, and dangerous (35). Terms such as evil (5) and beast (37) were also used in the headlines about the parricide in Bolzano. Parricide is often treated as a rare but horrific deed that is the result of severe mental illness or an abusive childhood (9). Parricide was once labeled “the schizophrenic crime” (38), but it soon became clear that it is much more complex (39). Vahabzadeh et al. (40) found that there was a decrease in reporting of crime committed by people with schizophrenia in 2010 compared with 2000. However, no significant decrease was found in metaphorical usage of the terms schizophrenia and schizophrenic (40). General attitudes toward mental health have improved in recent years (41, 42), but many people still hold stigmatized views. Negative newspaper coverage of mental illness and, in particular, violence committed by people with mental problems contribute to stigmatization (43). Among other things, demographic variables (e.g., age, gender) influence the attitudes toward mental illness (41). A common theme among research findings is the idea that people with mental illness, particularly schizophrenia, are dangerous and should be feared, which often leads to these people being met with avoidant behaviors (44–46). The factor “perceived dangerousness” is one of the determining factors for social distance from people with mental illness in various groups, e.g., the general population, psychiatric staff, relatives (44, 46, 47). The linking of mental illness with violence helps to perpetuate stigmatizing and discriminatory practices toward mentally ill people (35). Ross et al. (48) showed that negative news about psychiatric patients increases stigmatization toward them, while positive news decreases it.

With regard to the scientific findings on psychiatric patients and violence, we would first like to point out that the level of crime experienced by people with severe mental illness is higher than in the general population (49, 50). Studies on clinical populations indicate high rates of victimization among psychiatric patients [e.g., (51, 52)]. On the one hand, abuse can be a risk factor for psychiatric disorders; on the other hand, psychiatric disorders can also be a risk factor for victimization [e.g., (53, 54)]. It should also be noted that, for example, factors such as past violence and substance abuse are known to be significantly associated with aggression in psychiatric patients (55, 56). Mental illness may increase the likelihood of some individuals becoming violent, but only a small part of violence in society can be attributed to psychiatric patients (57). Muravyeva et al. (9) state that it is not only abused teenagers and mentally ill men living at home with their mothers who kill their parents. The American Psychological Association (58) notes in an article on mental illness and violence that a diagnosis alone cannot determine who may commit violent acts, and most people with mental illness are not violent. Nevertheless, prejudices persist, for example that people with mental illness are largely responsible for mass shootings and other acts of mass violence (58).

There are several studies examining the stigmatization of mental illness in different samples. In a comparative nationwide survey in Italy (59), in which lay respondents, professionals and relatives took part, the lay respondents differed from the other two groups in their opinion of the unpredictability of the patients: 85% stated that patients with schizophrenia are unpredictable; among the relatives, the rate was 64% and among the professionals 74%. An Austrian study with a representative sample of the general public, various professional groups and relatives (46) found that the general population had a more pessimistic view concerning all aspects of schizophrenia than relatives and staff members. In a study of 176 healthcare professionals from the Netherlands, Gras et al. (60) showed that general practitioners scored significantly higher on the Mental Illness Clinicians Attitude questionnaire which is used to assess stigmatizing attitudes than forensic mental health professionals and the latter scored significantly higher than mental health professionals.

The present study focused on the emotional reactions, attitudes and stigmatization of mental problems in the context of parricide: As stated above, (a) the Bolzano parricide received much media attention [e.g., (14, 19)], (b) the media in general tend to sensationalize parricides (9, 11) and associate violence and parricide with mental illness (9, 35), even though only a small proportion of violence in society can be ascribed to psychiatric patients (57). In the case of such a serious offense, the average citizen wondered whether anyone could become so evil, or whether the perpetrator must have a mental disorder [compare (61), p. 17]—and, conversely, that psychiatric patients must be feared as potential perpetrators. Based on this, the aim of this study was to analyze the emotional reactions to the parricide in Bolzano in January 2021 and the associated stigmatization of psychiatric patients in the South Tyrolean general population (persons with mental disorders, relatives of people with mental disorders and population with no direct or indirect contact with persons with mental disorders) and the staff of the Department of Psychiatry in Bolzano. To the best of our knowledge, the present study is the first to examine the emotional reactions and stigmatization of psychiatric illness in relation to parricide in a well-defined region and population. Based on the existing literature on crime (32, 33), we hypothesize that the parricide was associated with emotional reactions, particularly fear and anger. In addition, we hypothesize that the four different groups (persons with mental disorders, relatives, mental health professionals, population with no direct or indirect contact with mental disorders) are associated with different levels of emotional reactions and stigmatization. It is assumed that affected people, relatives, and mental health professionals report less stigmatization than the population with no direct or indirect contact to mental disorders [compare, e.g., (46, 59, 60)]. Furthermore, we hypothesize that different demographic variables, namely age and gender (41), are important factors for stigmatization, i.e., age and gender of the population are related to how people with mental health problems get perceived.

## 2 Methods and methodology

### 2.1 Setting and procedure

The study was conducted on the occasion of a parricide in South Tyrol, a northern Italian region with 533,715 inhabitants (62). The parricide occurred on January 4th 2021 (1), the court trial, which was again accompanied by increased media coverage of the case, between March 4th 2022 and December 19th 2022 (63).

The sub-study involving mental health professionals was conducted online from June to September 2022. All employees of the Department of Psychiatry of Bolzano (Italy), working in both in-patient (i.e., acute hospital, psychiatric rehabilitation, psychiatric communities) and out-patient (territorial mental health centers) services, were invited to participate in the study by email. The Department of Psychiatry comprises a total of 220 employees.

In addition, from January to March 2023, the general population of South Tyrol (Italy) was invited to participate in the online study. The study link was distributed via the local media (online as well as in printed formats).

The minimum age for participation was 18 years. Participants gave their written informed consent. They could withdraw from further participation at any stage of the survey without giving a reason. The study was approved by the ethics committee of the Azienda Sanitaria dell’Alto Adige (Engl. Medical District of South Tyrol; No. 111–2022 in date 16th November 2022).

### 2.2 Participants

Our study included *N* = 2,262 South Tyrolean participants (1,536 females; 67.90%) divided into four groups: *n* = 267 persons with mental disorders (183 females; 68.54%), *n* = 855 relatives of persons with mental disorders (632 females; 73.92%), *n* = 121 mental health professionals (87 females; 71.90%) and *n* = 1,019 persons with no direct or indirect contact to mental disorders (634 females; 62.22%). Table 1 shows the sociodemographic variables, e. g. the language group (German, Italian, Ladin, other), of the persons with mental disorders, relatives and persons with no direct or indirect contact to mental disorders. Mental health employees had the following characteristics: 17 psychiatrists (14.05%), 15 psychologists (12.39%), 50 nurses (41.32%), 9 specialized therapists (e. g. occupational therapy, psychiatric technicians; 7.44%), 7 social workers (5.79%) and 19 other employees (15.70%) took part in the survey. They work in different areas of the Department of Psychiatry of Bolzano: 26 in the acute care unit of the hospital (21.49%), 40 in a territorial center for mental health (33.06%), 42 in a center for psychiatric rehabilitation (43.71%), 4 in psychiatric communities (3.31%) and 9 in other services of the Department of Psychiatry of Bolzano (7.44%). Most of the mental health professionals (*n* = 38; 31.40%) have been working in psychiatry for over 20 years (total professional experience in psychiatry: 0–5 years: *n* = 37 (30.58%); 6–10 years: *n* = 14 (11.57%); 11–15 years: *n* = 15 (12.40%); 16–20 years: *n* = 17 (14.05%)). In terms of age, most mental health professionals (*n* = 59; 48.76%) were between 50 and 60 years old (20–30 years: *n* = 11 (9.09%); 30–40 years: *n* = 13 (10.74%); 40–50 years: *n* = 26 (21.49%); over 60 years: *n* = 12 (9.92%)).

**Table 1:** Sociodemographic variables of the sample.

	Persons with MD	Relatives	Persons without contact	Total
Age	*M* = 44.83 (*SD* = 14.96)	*M* = 46.33 (*SD* = 13.74)	*M* = 48.88 (*SD* = 14.48)	*M* = 46.68 (*SD* = 14.39)
Level of education				
Primary school Middle school High-school diploma Apprenticeship Bachelor degree Master degree	5 (1.87%) 19 (7.12%) 91 (34.08%) 52 (19.48%) 44 (16.48%) 56 (20.97%)	1 (0.12%) 43 (5.03%) 244 (28.54%) 148 (17.31%) 147 (17.19%) 272 (31.81%)	9 (0.88%) 64 (6.28%) 337 (33.07%) 176 (17.27%) 143 (14.03%) 290 (28.46%)	15 (0.70%) 126 (5.86%) 672 (31.39%) 376 (17.56%) 334 (15.60%) 618 (28.87%)
Language group				
German Italian Ladin Other	197 (73.78%) 56 (20.97%) 10 (3.75%) 4 (1.49%)	610 (71.35%) 217 (25.38%) 24 (2.81%) 4 (0.47%)	729 (71.54%) 250 (24.53%) 38 (3.73%) 2 (0.19%)	1,536 (71.74%) 523 (24.43%) 72 (3.36%) 10 (0.47%)

MD, mental disorders; M, mean; SD, standard deviation.

### 2.3 Measures

The online survey was provided in German and Italian, since the study was conducted in South Tyrol, an autonomous province in Northern Italy, where 69.4% of the population belongs to the German, 26% to the Italian and 4.5% to the Ladin language group (62).

#### 2.3.1 Sociodemographic variables

Participants reported their gender, age, level of education and language group. In addition, they were asked whether they suffer from a mental illness and whether their relatives or close friends suffer from a mental illness. Furthermore, employees of mental health services provided information on their years of professional experience and their years of professional experience in the psychiatric field.

#### 2.3.2 Emotional reactions in the context of the parricide in Bolzano

Participants were asked what impact the parricide had on their emotional state. They could choose between sadness, anger, disgust, fear and indifference. In the questionnaire for mental health professionals, the participants had to select one answer (single-choice); in the other versions (people with mental disorders, relatives and people with no direct or indirect contact) multiple answers were possible.

#### 2.3.3 Perceived dangerousness of psychiatric patients

Participants were asked: “Do you believe that psychiatric patients are at greater risk of committing parricide?” Mental health professionals could answer with “no” or “yes.” The other participants could give their answer on a 4-point Likert scale (“never”—“often”).

#### 2.3.4 Reported and intended behavior scale

The *Reported and Intended Behavior Scale* [RIBS; (64)] is a psychometrically tested instrument to assess reported (past and current) and intended (future) behavioral discrimination among the general public against people with mental health problems. It inquires about reported and intended behavior among four different contexts: (1) living with, (2) working with, (3) living nearby and (4) continuing a relationship with someone with a mental health problem. The questionnaire consists of eight items (e.g., “Are you currently working with, or have you ever worked with, someone with a mental health problem?”). The first four items are designed to assess the prevalence of behavior in each of the four contexts (responded with “yes” or “no” or “do not know”) whereas items 5–8 ask about intended behavior within the same contexts [responded on a 5-point-Likert scale from “agree strongly” to “disagree strongly” plus “do not know”; (64)]. For the Italian version of the questionnaire, we used the psychometrically validated Italian version of the RIBS (65). For the German version of the questionnaire, we back-translated the Italian version (65).

### 2.4 Data analysis

We carried out descriptive statistics. In addition, we used one-way Anovas with Tuckey’s method *post-hoc* tests and one-way Welch tests with Games Howell *post-hoc* tests to test group differences. The Levene test was used to test homogeneity of variance. In order to examine whether the general openness toward people with mental disorders (total RIBS score) predicted beliefs about parricide, we carried out regressions.

*p*-values ≤5% were considered significant. R Studio version 2022.07.2 was used for data analysis.

## 3 Results

### 3.1 Emotions experienced in the context of the parricide

**Figure 1:**
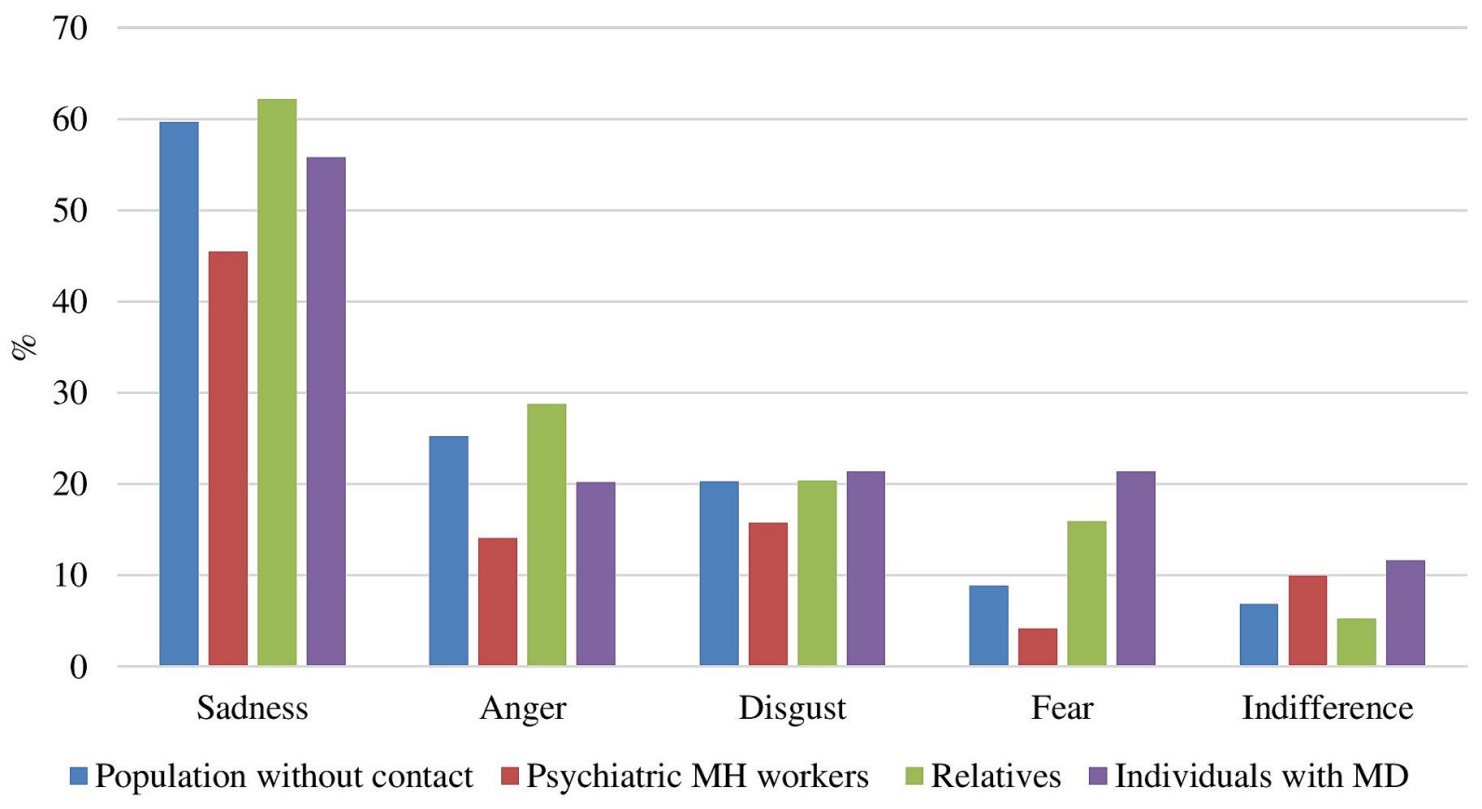
Emotions experienced by persons with mental disorders, relatives, persons with no direct or indirect contact and mental health professionals. MD, mental disorders; MH, mental health.

Figure 1 displays the percentage of the emotions experienced by the different groups. The emotion all four groups experienced the most was sadness: 62.22% of relatives, 59.67% of non-contact persons, 55.81% of affected persons and 45.45% of the mental health professionals reported experiencing sadness in the context of the parricide. Relatives were the group which experienced most sadness (62.22%), while mental health professionals felt the least sadness (45.45%). This same pattern was found for anger: relatives experienced most anger (28.77%), mental health professionals the least (14.05%). Regarding fear, people with mental disorders (21.35%) and relatives (15.91%) experienced more fear than the general population (8.83%) and mental health professionals (4.13%).

### 3.2 Psychiatric patients at a greater risk of committing parricide?

80.17% of mental health professionals responded that psychiatric patients are at no greater risk of committing parricide than the general population. 19.83% stated that the psychiatric patients are at a greater risk.

**Figure 2:**
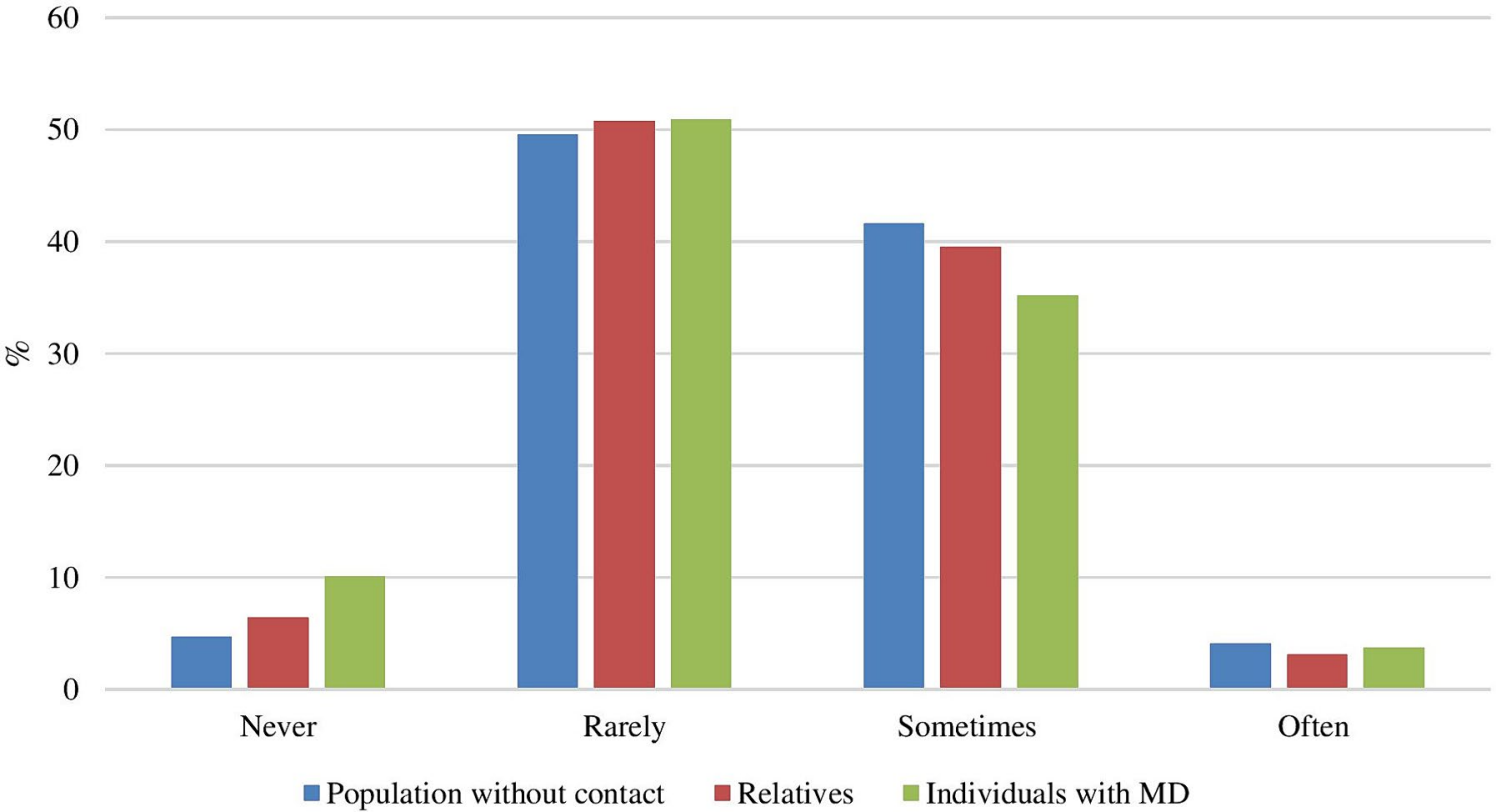
Do you believe that psychiatric patients are at greater risk of committing parricide? MD, mental disorders.

Furthermore, a significant group difference regarding the perceived risk was found [*F*(2, 2,132) = 4.62, *p* < 0.01]. The population with no contact (*M* = 1.45, *SD* = 0.65) was significantly more likely than those with mental disorders (*M* = 1.33, *SD* = 0.71) to believe that psychiatric patients are at greater risk of committing parricide (*p adj*. < 0.05). Figure 2 shows the percentage results. In addition, age was found to be significant [*F*(1, 923) = 15.56, *p* < 0.001]: older age correlated weakly but significantly with a lower perceived risk for psychiatric patients to commit parricide [*r* = −0.08, *t*(2,135) = −3.59, *p* < 0.001]. The gender of the participants did not yield significant results (*p* = 0.59).

### 3.3 RIBS—reported and intended behavior toward people with mental disorders

Table 2 shows the four different contexts (living with, working with, living in the neighborhood of, maintaining a relationship with someone with a mental health problem) of the RIBS questionnaire with its means and standard deviations for the four groups. The higher the mean score in the respective domain, the higher the openness of this group to live, work, have as a neighbor or continue a friendship with a person with a mental health problem.

**Table 2:** Means (SD) of the RIBS questionnaire in the four groups.

	Living	Working	Living nearby	Relationship	RIBS-total score
Population without contact	2.55 (1.13)	3.18 (1.22)	3.19 (1.25)	3.32 (1.31)	12.2 (4.14)
Relatives	2.92 (1.25)	3.56 (1.20)	3.56 (1.25)	3.74 (1.3)	13.8 (4.22)
Persons with MD	3.27 (1.28)	3.73 (1.18)	3.79 (1.21)	3.90 (1.24)	14.7 (4.24)
MH professionals	2.93 (1.21)	3.99 (0.09)	3.71 (1.22)	4.16 (1.3)	14.8 (3.37)

MD, mental disorders; MH, mental health.

#### 3.3.1 Openness to living with a person with a mental problem

Results show an overall significant group difference [*F*(3, 2,258) = 32.21, *p* < 0.001]. There is a significant difference between all groups except between relatives and mental health professionals (*p* = 0.99). As illustrated in Table 2, persons with mental disorders show the highest openness to live with a person with a mental problem followed by the mental health professionals and the relatives. The general population shows the lowest openness to live with a person with a mental health problem.

#### 3.3.2 Openness to working with a person with a mental problem

The results show a significant group difference in the reported and intended openness in working with persons with a mental health problem [*F*(3, 468.65) = 38.02, *p* < 0.001]. Regarding working with people with mental disorders, mental health professionals (*M* = 3.99, *SD* = 0.09), together with people with mental disorders (*M* = 3.73, *SD* = 1.18) show the highest scores (*p* < 0.001), followed by the relatives (*M* = 3.56, *SD* = 1.20, *p* < 0.001). Here again, the population without contact has the lowest score in regard to openness to working with a person with a mental health problem (*M* = 3.18, *SD* = 1.22, *p* < 0.001).

#### 3.3.3 Openness toward a neighbor with a mental problem

There is a significant difference between the groups in terms of openness to having a neighbor with a mental health problem [*F*(3, 2,258) = 24.74, *p* < 0.001], except for the mental health professionals that do not differ significantly from the persons with mental disorders and the relatives (see Table 2 for *M* and *SD*). Persons with mental disorders (*M* = 3.79, *SD* = 1.21) show the highest score whereas the population without contact shows the lowest score (*M* = 3.19, *SD* = 1.25).

#### 3.3.4 Openness to continuing a friendship with a person with a mental problem

**Figure 3:**
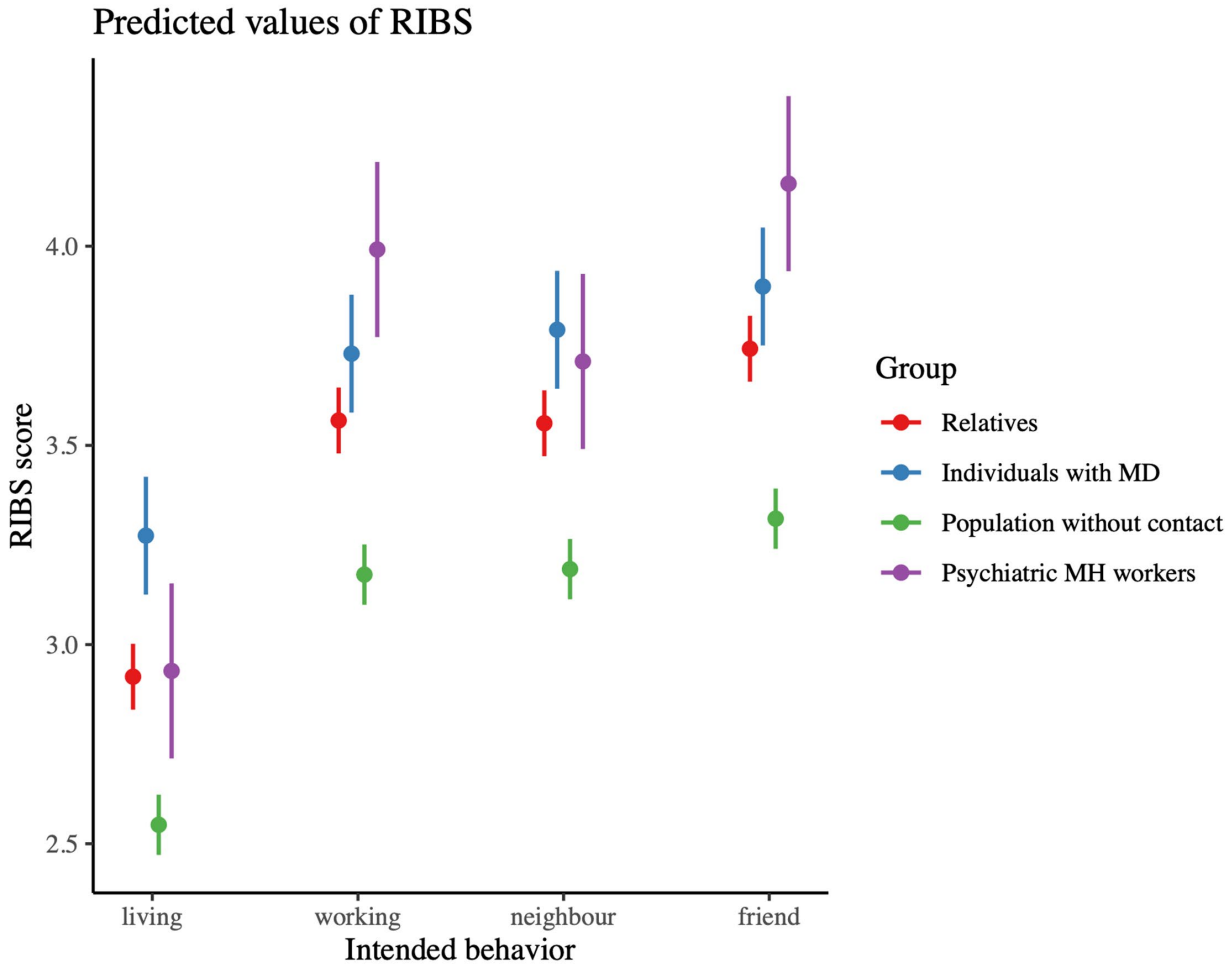
Intended behaviors of the four groups. MD, mental disorders; MH, mental health.

There are significant group differences regarding the openness to continue a friendship with a person with a mental health problem [*F*(3, 468.65) = 36.421, *p* < 0.001], with the exception of mental health professionals, who do not differ significantly from people with mental disorders and relatives (see Table 2 for *M* and *SD*). As visualized in Figure 3, mental health professionals (*M* = 14.8, *SD* = 3.37) have the highest score and the population without contact (*M* = 12.2, *SD* = 4.14) the lowest score.

#### 3.3.5 RIBS total score

The results show a significant group difference in the total RIBS score [*F*(2, 2,133) = 54.24, *p* < 0.001]. There are significant differences between all groups, with the exception of persons with mental disorders and mental health professionals, who do not differ statistically in their openness toward people with mental disorders, with the latter also not differing from the relatives. Also age was found to be significant [*F*(1, 2,133) = 76.09, *p* < 0.001] with a negative correlation (*r* = −0.20) showing that older age is associated with lower RIBS scores [*t*(2,136) = −9.59, *p* < 0.001]. No significant effect of gender on the RIBS total score was found (*p* = 0.39).

#### 3.3.6 Relationship between openness toward people with mental disorders and belief in parricide (only for people with mental disorders, relatives, and population with no contact)

We found that the RIBS significantly predicted the perception that psychiatric patients have a higher risk of committing parricide (*β* = − 0.047, *SE* = 0.008, *t* = −5.81, *p* < 0.001). No interaction effects were found with type of group, gender or age.

## 4 Discussion

The aim of this study was to investigate the emotional reactions of the South Tyrolean population to the parricide in Bolzano (South Tyrol, Italy) in January 2021, in different target groups (mental health professionals and general population, including people with mental health problems, relatives and persons with no direct or indirect contact). Moreover, we investigated the stigmatization of psychiatric patients in the context of parricide.

In terms of emotional reactions to parricide, all groups were most affected by sadness. Emotional reactions to crime are a somewhat peripheral topic within theoretical criminology (66) and have been under-researched (32). In their article on emotional reactions to crime in different cultures, Matsumoto and Hwang (67) reported that there is little reason to believe that crime does not elicit negative reactions including anger and sadness, although there are likely to be cultural differences in the absolute level of emotions experienced. A qualitative Portuguese study (68), which aimed to understand how the media shapes the fear of crime, showed that it was not possible to establish a clear relationship between media consumption and feelings of insecurity, and that the media does not totally shape the fear of crime, as a variety of meanings have emerged, that differentiate its influence. Sensationalism, the location of the reported crime and the realism of the news are factors of great relevance (68). Pantti and Wieten (69) showed that mourning was the main emotional reaction to the murder of a Dutch right-wing politician and titled their study “Mourning becomes the nation”. On the night of the murder, a Dutch Journal opened with “What no one thought would be possible in the Netherlands, did happen after all”, and such messages were repeated again and again. Pantti and Wieten (69) demonstrated in their study how the media coverage of the murder created a binding element in the Dutch population by focusing on mourning and converting emotions like anger and hatred into a unifying and less destructive narrative, namely grief. Another explanation for the fact that all groups experienced sadness the most in connection with the parricide of Bolzano could be linked to the findings of Rhyderrch (70): compared to 2008, there were more anti-stigmatizing articles in the English media in 2014 and a significant decrease in the proportion of articles featuring the stigmatizing elements “danger to others” and “personal responsibility” as well as an increase in “hopeless victim”. Cultural differences must be taken into account, as parricide is also referred to as a culturally sensitive phenomenon (9). Boots and Heide (7), in a content analysis of available media reports on parricide in different cultures, found that the sample of parricide cases (*n* = 76) not from the U.S. (United States) was relatively small. They argued that this may be partly due to reading reports in English-language online sources, but also the fact that parricide simply does not occur in some countries, perhaps, because of religious or cultural reasons. It could also suggest that it does not reach the public because it is taboo, offensive, and the government exercises tight control over the reporting of such events. Furthermore, the authors argued that the greater reported incidence of abuse as an underlying dynamic in U.S. parricide cases compared to non-U.S. cases may reflect an increased sensitivity to the existence of abuse in the U.S., rather than actual differences in the incidence of abuse across cultures, as significant attention has focused on abuse in the U.S. since the 1960s (7). Cultural differences in parental authority, obedience, disciplinary and punishment practices as well as generational conflicts and how they affect modern families in different parts of the world, may influence the dynamics of parricides (e.g., the absence of self-defensive killings in Asian societies in contrast to European and North American societies), but also how parricide is seen and judged (9).

Comparing the groups, in the present study the relatives experienced more sadness and anger than the other groups. This could be related to the fact that in families in which a member has a mental disorder there are times when a high level of emotional involvement is required (71). The high level of emotional involvement of relatives (72) and their burden [e.g., (73)] could be possible explanations as to why relatives reported more sadness and anger than the other groups. The burden of relatives tends to be underestimated. For example, in a study with 48 relatives of patients with schizophrenia or bipolar disorder and 39 mental health professionals, the professionals underestimated the burden of symptoms experienced by relatives of patients with bipolar disorders (74).

With regard to the risk of psychiatric patients committing parricide, over 80% of mental health professionals in the present study responded that psychiatric patients were at no greater risk than the general population. This high percentage may be due, at least in part, to the fact that a fundamental stigma-reducing strategy is education about stigmatization and the damage it can do to clients’ confidence, hope, recovery, and life chances (75), which is part of the education and training of mental health professionals. The non-contact population was more likely than the affected population to believe that psychiatric patients were at greater risk of committing parricide. In fact, one strategy to reduce stigmatization is interpersonal contact with people with mental illness. In a review of the literature on interpersonal contact and the stigmatization of mental illness, Couture and Penn (76) stated that both retrospective and prospective contact tended to reduce stigmatizing views of people with mental illness.

The results in relation to the RIBS score also point in the same direction: the non-contact population showed the least openness toward people with mental health problems. This applied to all four categories of the RIBS: living, working, having a neighbor, and continuing a friendship with a person who has mental health problems. Our results are in line with results from Italy (59) and Austria (46). The representative Austrian results are consistent with our findings in that the general population has a more pessimistic view (in the Austrian study regarding aspects of schizophrenia; in our study regarding openness toward people with mental health problems) than relatives and staff members (46). Our study confirms that the willingness to socialize with patients with mental disorders strongly depends on the closeness of such contact (46), and extends this knowledge to the context of parricide. It is assumed that education and knowledge, increased mental health awareness as well as social contact promote familiarity with mental illness, which can reduce stigmatization and prejudicial attitudes (77–79).

As far as demographic variables are concerned, there were no differences between the genders in our study, but there were age differences: younger people were more likely than older people to say that psychiatric patients are at a higher risk of committing parricide than the general population, and they were less open toward psychiatric patients. Women are often reported to be more understanding, less fearful and less likely to marginalize the mentally ill than men (e.g., (80)), although there are also articles that found no differences between the genders [e.g., in the case of schizophrenia in Bradbury (41) and Crisp et al. (81)]. A study from the United Kingdom found that 70% of respondents consider people with schizophrenia to be dangerous and unpredictable. In this study, no significant differences were found in terms of age and gender (81). In an article by Bradbury (41), the results showed that both age and gender influenced attitudes toward generalized anxiety disorder (a common mental health condition), but not toward schizophrenia (a complex mental illness). A systematic review of population studies on gender differences in public beliefs and attitudes about mental disorder in Western countries (82) showed that women do not appear to have a more positive attitude toward people with mental disorders than men. In terms of age, although young people have grown in a society where mental illness is discussed publicly at school and in the media, in the present study younger people were more likely than older people to say that psychiatric patients were at higher risk of committing parricide. Bradbury (41) also found that the over-40 years group consistently reported lower levels of stigmatizing attitudes than the 16–18 age group. The author explains this result with the meta-analyses by Corrigan et al. (83) and Grifths et al. (84): older people are more likely to have already had sustained contact with a person with a mental health diagnosis, which can potentially reduce stigmatizing attitudes. Moreover, young people may be more stigmatizing due to factors such as social pressure to behave and appear as a well-functioning member of their peer group (85). Jorm and Wright (86) reported that young people are more inclined to view mental illness as a personal failure or weakness rather than a genuine health issue. According to Bradbury (41), this result also supports the works of Gonzalez et al. (87) and of the TNS-BMRB (88), which suggest that, as people get older, they are more informed about and accept people who are different from themselves. Another explanation could lie in the fact that younger people use more social media, which repeatedly stigmatize and trivialize mental health conditions (e.g., in comparison to physical conditions) (89).

### 4.1 Practical implications

The findings provide indications of factors that can be helpful in combating the stigmatization of mental illness. The results of the study indicate that personal contact with people with mental health problems and appropriate information and education are associated with less stigmatization of people with mental health problems. These results should be taken into account both in the field of mental health (education and training) and in the wider population (school education, media, etc.). In fact, three main approaches are known that can be used to reduce the stigmatization of people with mental illness: contact, education, and protest or social activism (90), with the first two approaches having been extensively evaluated (83). Furthermore, the media play an important role in this context. Local media and social media should be used to disseminate reputable, expert-validated information to counteract simplistic, stigmatizing, trivializing, and careless conclusions [e.g., (89)]. Regarding the results on emotional reactions, which showed that relatives experienced more sadness and anger than the other groups, it is important to point out that this group in particular needs support in the form of psychological and peer assistance. To summarize, there is already a certain level of awareness and sensitivity in THE South Tyrolean society with regard to mental illness (e.g., relatively appropriate emotional reactions to parricide, the majority of the population does not believe that psychiatric patients have an increased risk of committing parricide), which is due to the good work done in this regard in South Tyrol in recent years (see, e.g., campaigns and presentation by the psychiatric services and the “Forum Prävention”). Nevertheless, based on the results of this study, we must continue and intensify our efforts at various levels in society and in research to further combat the stigmatization of mental illness. Our neighboring region of North Tyrol (Austria), for example, recently launched a university course for teachers aimed at destigmatizing mental illness in schools. From spring 2024, a pilot project teaching “Mental Health” in schools was implemented in North Tyrol (Land Tirol, 2023). The expansion of such and similar programs should also be tackled for our region.

### 4.2 Limitations and future research

This study also has some limitations. Firstly, mental health professionals (June–September 2022) and the other groups (January–March 2023) were surveyed in different time periods and with slightly different questionnaires (the response formats in terms of emotional reaction and perceived dangerousness differed). The different media coverage of the parricide at different time intervals from the parricide itself and at different stages of the court proceedings may have influenced the findings: the legal processing of the parricide started in March 2022 (91); the sentence of life imprisonment was pronounced on November 19th, 2022 (92). Secondly, the group that reported having no contact with people with mental health problems may have underlying biases (e.g., living in a social environment where mental health issues are denied, being oblivious or dismissive of mental health problems) that possibly influenced the outcomes. Moderator and mediator effects should be investigated in further research. Thirdly, the questions on the emotional reaction in the context of the parricide and the perceived dangerousness of psychiatric patients were not taken from standardized measurements. In addition, it was not possible to verify multiple answers from a single participant. It should also be mentioned that the comparison between people with mental disorders and relatives on the one hand and professionals and people with no contact on the other can be problematic to a certain extent, as the latter two groups think of mental disorders in general terms, while those affected and their relatives think of specific persons. In further research, this problem could be partially overcome by using case vignettes. Furthermore, it must also be taken into account that only people who were capable of completing an online questionnaire (cognitive ability, language skills, internet access) were able to participate in the study. Finally, although the study addresses a global issue, its findings are likely to be influenced by local mental health policies and mental health care organizations. Further studies are needed to compare the situation in South Tyrol with the situation in the rest of Italy and in other countries where mental health care is organized differently.

## Data availability statement

The raw data supporting the conclusions of this article will be made available by the authors, without undue reservation.

## Ethics statement

The studies involving humans were approved by the Ethics Committee of the Azienda Sanitaria dell’Alto Adige (Engl. Medical District of South Tyrol). The studies were conducted in accordance with the local legislation and institutional requirements. The participants provided their written informed consent to participate in this study.

## Author contributions

MS: Conceptualization, Data curation, Formal analysis, Investigation, Methodology, Visualization, Writing – original draft, Writing – review & editing. AW: Conceptualization, Methodology, Writing – original draft, Writing – review & editing. AO: Conceptualization, Investigation, Methodology, Project administration, Writing – review & editing. IG: Conceptualization, Investigation, Methodology, Writing – review & editing. FM: Conceptualization, Investigation, Methodology, Writing – review & editing. DB: Conceptualization, Methodology, Supervision, Writing – review & editing. AC: Conceptualization, Investigation, Methodology, Project administration, Supervision, Writing – review & editing.
